# The Frequency of Primary Sjogren’s Syndrome and Fibromyalgia in Irritable Bowel Syndrome

**DOI:** 10.12669/pjms.331.11168

**Published:** 2017

**Authors:** Funda Erbasan, Yesim Cekin, Deniz Turgut Coban, Ugur Karasu, Dinc Suren, Ayhan H. Cekin

**Affiliations:** 1 Funda Erbasan, MD. Specialist, Department of Rheumatology, Antalya Training and Research Hospital, Antalya, Turkey; 2 Yesim Cekin, MD. Associate Professor, Department of Microbiology, Antalya Training and Research Hospital, Antalya, Turkey; 3 Deniz Turgut Coban, MD. Specialist, Department of Ophthalmology, Antalya Training and Research Hospital, Antalya, Turkey; 4 Ugur Karasu, MD. Specialist, Department of Rheumatology, Antalya Training and Research Hospital, Antalya, Turkey; 5 Dinc Suren, MD. Associate Professor, Department of Pathology, Antalya Training and Research Hospital, Antalya, Turkey; 6 Ayhan H. Cekin, MD. Associate Professor, Department of Gastroenterology, Antalya Training and Research Hospital, Antalya, Turkey

**Keywords:** Irritable Bowel Syndrome, Sjogren’s Syndrome, Fibromyalgia, Antinuclear antibodies

## Abstract

**Objective:**

To determine the frequency of sicca complex, Sjogren’s Syndrome (SS) and Fibromyalgia (FM) in patients with Irritable Bowel Syndrome (IBS).

**Methods:**

Seventy seven IBS patients who fulfilled the Rome-III criteria were included in the study. All patients were assessed for FM according to the American College of Rheumatology (ACR) 2010 criteria. After examination for objective evidence of sicca complex by Schirmer test, TBUT and Ocular Staining Score (OSS), serological tests were performed. And the diagnosis of SS was made according to the American College of Rheumatology (ACR) classification criteria for SS – 2012.

**Results:**

Thirteen (16.9%) of IBS patients had FM. Dry eye was detected in 20(26.0%), 7(9.1%) and 29(37.7%) patients by OSS, Schirmer test and TBUT, respectively. Of 77 patients with IBS, the diagnosis of SS was established in two patients (2.6%).

**Conclusion:**

The frequency of Sjogren’s Syndrome among patients with IBS is relatively higher than the general population. All IBS patients should be questioned for dryness of the mouth and eyes, and if necessary, should be evaluated for SS.

## INTRODUCTION

Sjogren syndrome (SS) is a systemic autoimmune disease characterized by lymphocytic infiltration and destruction of exocrine glands associated with dryness of eyes and/or mouth. In addition to the exocrine glands, many other organs such as lungs, muscles and gastrointestinal system can be involved in patients with SS. It is known that a great number of these patients suffer from functional bowel symptoms, dysphagia and impaired gastric emptying rate.^1^-^3^ The prevelance of irritable bowel syndrome (IBS) was reported as 39% in SS.^1^ In addition to extraglandular features and sicca complaints, fibromyalgia (FM) and subjective symptoms, such as fatigue and pain, can be seen in patients with SS.

FM is a common cause of chronic widespread musculoskeletal pain which is often accompanied by fatigue, cognitive disturbance and psychiatric symptoms. Among many other symptoms and signs, a substantial portion of patients with FM present concomitant symptoms of ocular and oral dryness.^4^ FM is often present in patients with other common functional somatic syndromes (FSS), including chronic fatigue syndrome, temporomandibular disorder and IBS.^5^ Up to 81% of patients with a diagnosis of FM have symptoms of IBS.^6^

IBS is a gastrointestinal syndrome characterized by chronic abdominal pain and altered bowel habits in the absence of any organic cause. Patients with IBS often suffer from a broad range of non-gastrointestinal symptoms, such as dysmenorrhea, dyspareunia, increased urinary frequency and urgency, and FM.^7^ Also sicca complex can be seen in 33% of IBS patients.^8^

Patients with IBS frequently have FM and sicca symptoms, but it is unknown whether these patients are a subgroup of the SS. The frequency of SS in IBS has not been evaluated so far. The aim of the present study was to determine the frequency of fibromyalgia, sicca symptoms and SS in patients with IBS based on the diagnostic criteria of the American College of Rheumatology (ACR). We think that this study can provide information about the need for evaluation for SS in patients with IBS.

## METHODS

The study cohort included consecutive patients with IBS who were under surveillance at the Department of Gastroenterology in Antalya Training and Research Hospital between 2012 and 2013. The patients with abdominal pain and altered bowel habits were investigated by appropriate laboratory samples; followed by radiological and/or endoscopic examination when clinically indicated. Patients with gastrointestinal symptoms without any organic disease, who fulfilled the Rome-III criteria, were classified as IBS.^9^ The study protocol was approved by the Ethical Committee of Antalya Training and Research Hospital. All patients gave informed consent before participation.

All patients were assessed to identify the presence of FM according to the American College of Rheumatology (ACR) 2010 criteria (Widespread pain index and symptom severity scale).^10^ The diagnosis of SS was defined according to the American College of Rheumatology (ACR) classification criteria for SS – 2012.^11^

All patients were asked about their other systemic diseaeses and drugs that may affect salivary or lacrimal secretion. The patients with a history of human immunodeficiency virus, hepatitis C, amyloidosis, sarcoidosis, active tuberculosis, graft versus host disease, or autoimmune connective tissue diseases; previous head and neck radiation treatment; current treatment with daily eye drops for glaucoma; corneal surgery within prior five years to correct vision; cosmetic eyelid surgery within prior five years; and contact lens wearers were not included in the study. All patients underwent a complete ophthalmic evaluation. And then Schirmer test, tear film tear break-up time (TBUT), corneal epithelial staining using fluorescein and conjunctival epithelial staining using lissamine green were performed to detect dry eye. The eyes were graded separately and the Ocular Staining Score (OSS) recorded. Schirmer<5 mm (without anesthesia), TBUT of ≤10 seconds or OSS ≥3 were considered as abnormal and a sign of KCS.

Antinuclear antibodies (ANA) were detected by indirect immunofluorescence (IIF) on HEp-2 cells (Euroimmun, Germany). ANA profile test was performed using ANA profile 3 kit (Euroline, Euroimmun, Germany) at 1/100 dilution in accordance with the manufacturer’s recommendations. Rheumatoid factor (RF) was detected with the commercially available kit (N Latex RF kit, Siemens Diagnostics, Germany).

When clinically indicated minor salivary gland biopsy was taken just after taking their permission. Biopsy were immediately fixed in 10% formaldehyde and embedded in paraffin. Then, 4-μm thick sections were obtained from paraffin blocks and were stained with hematoxylin-eosin for conventional histopathological examination. The focus score (FS) was defined as the group of inflammatory cell aggregates containing at least 50 mononuclear cells per 4mm2 of tissue area.^12^

The data were analyzed with descriptive statistics. Data are presented as Mean ± SD or percentage. Statistical analyses were performed using SPSS software version 18.

## RESULTS

Overall 77 patients (66 women and 11 men) were included to the study. Epidemiological and clinical features of the study population are shown in Table-I.

**Table-I:** Epidemiological and clinical features of the study population n(%).

Features	Patients n:77
Female/male	66/11
Age, mean±SD, yrs	39.7±9.48
Dry mouth symptoms	29(37.7)
Dry eye symptoms	27(35.1)
Dry eye in female/male	23/4
Raynaud’s phenomenon	3(3.9)
Co-morbid diseaes	
Autoimmune thyroiditis	1(1.3)
Diabetes Mellitus	3(3.9)
Hypertension	2(2.6)
Depression	12(15.6)

Twenty-seven (35.1%) patients with IBS had dry eye symptoms. Dry eye was detected by Schirmer test in 7 (9.1%) patients, by TBUT in 29 (37.7%) and by OSS in 20 (26.0%) patients. However, 9 (11.7%) patients were using systemic medications that could be contributing to their dry eye syndrome. These included 8 patients (10.4%) using antidepressants and one patient (1.3%) using anticholinergics.

Thirteen (16.9%) of IBS patients had FM. Eigth of these patients had dry eye symptoms, and four met the criteria for clinically significant dry eye. Nine patients (11.7%) had dry mouth and ANA was positive only in three patients with FM. No statistical difference was found in dry eye findings between male and female patients.

All samples were negative for RF. Twelve patients (15.6%) were positive for ANA. In 8 of these 12 patients, there was no clinical evidence or sign of SS other than low titre (≤1:100) ANA positivity. Moreover, these patients were all negative for anti-SSA, anti-SSB and other autoantibodies which were analyzed. Clinical findings were present in remaining four patients with positive ANA; of these, SS-A antibodies were positive in 2 patients. The autoantibodies such as anti-SSB, anti-U1 RNP, anti-Sm, anti-scl-70 and the others were all negative in these patients. Of these, one patient with negative ANA had anti-SSA with no symptom or sign.

Of 20 patients with OSS≥3, ANA was found to be positive in four patients whereas anti-SSA was found to be positive in 2 patients. In remaining 16 patients, all auto-antibodies were negative. Of these, there was systemic drug use in 5, diabetes mellitus in two and five, pterygium in 3 and meibomian gland dysfunction in 1). Thus, no biopsy was performed in these patients who had no symptom other than dry eye.

There were four patients with suspected SS; however, biopsy was performed in two patients. One patient refused the biopsy just before procedure and the other one was out of the region. Salivary gland inflammatory focus score was one (FS=1) in both. The clinical details of these patients are shown in Table-II. Of 77 patients with, the diagnosis of SS was established in only two (2.6%) patients.

**Table-II:** Clinical characteristics and laboratory findings of patients with suspected SS.

	Patient 1	Patient 2	Patient 3	Patient 4
Sex	Female	Female	Female	Female
Age	51	38	46	28
Fibromyalgia	+	-	+	-
Dry eye symptoms	+	+	+	+
Dry mouth symptoms	+	+	+	+
Arthralgia	+	-	+	+
RP	-	-	-	-
Schirmer<5 mm	+	+	+	+
TBUT ≤10 sec	+	+	+	+
OSSOSS≥3	+	+	+	+
RF	Negative	Negative	Negative	Negative
ANA	Speckled	Speckled	Speckled	Speckled
ANA titre	1/100	1/320	1/320	1/320
ENAA	Anti-SSA	Anti-SSA	Negative	Negative
MSGB(FS)	One	One	- *	- *

SS: Sjogren’s syndrome, RP: Raynaud’s phenomenon, TBUT: Tear film tear break-up time, OSS: Ocular staining score, RF: Rheumatoid factor, ANA: Antinuclear antibodies, ENAA: Extractable nuclear antigen antibodies, MSGB: Minor salivary gland biopsy, FS: Focus score

* No biopsy was performed.

## DISCUSSION

Irritable bowel syndrome (IBS) is the most commonly diagnosed gastrointestinal condition and affects 7% to 21% of the general population.^13^ Sjogren’s syndrome is one of the most common autoimmune disorders, and the reported prevalence rates in population-based studies have varied from 0.03% to 2.1%.^14^-^16^ In this study, we found the frequency of SS in patients with IBS as 2.6%. The prevalence rates can vary considerably depending on the classification criteria used, study design, and the population examined. The published data on incidence and prevalence of SS are based on studies using the Copenhagen criteria, European classification criteria and American–European consensus group (AECG) criteria. In this study we used the ACR-2012 classification criteria and evaluated a relatively small patient group, these may be the reason for the high frequency found in the present study.

Dry eye is one of the key clinical features and possibly an early clinical presentation of SS. It has been reported that approximately 4.8% of the patients with dry eye may develop SS in about 3.8 years.^17^ Patients with IBS and FM have similar complaints with SS, such as dry eye and dry mouth. Prevalence estimates of dry eye disease and symptoms have largely varied by study, ranging between 11-38% in normal population.^18^,^19^ Previous studies have reported a wide range of sicca symptoms varying from 18-71% in FM patients and 33% in IBS patients.^4^,^8^,^20^ While dry eye symptoms were found in 27 patients with IBS in our study, only 20(26.0%), 7(9.1%) and 29(37.7%) patients had clinically significant dry eye by OSS, Schirmer test and TBUT, respectively.

However, there were systemic conditions such as diabetes mellitus or local diseases such as pterygium, pingeucula or meibomian gland dysfunction in seronegative patient with dry eye. FM was found in only one of 15 patients with dry eye. Therefore, we couldn’t establish a relationship between eye dryness and FM. Dry eye has a complex and multifactorial etiology. The mucosal dryness in FM is accepted most likely secondary to sympathetic hyperactivity, but there is no information about the cause of dry eye in IBS.^21^ Inflammatory infiltration of the lacrimal glands leading to cell death and tear hyposecretion is the cause of dryness in two patients who diagnosed as SS. However, the causal relationship between these two situations in other IBS patients is unclear. Furthermore, it has been reported that there is a female preponderance in terms of dry eye prevalence in IBS patients but the results of our study did not confirm this finding, because the dry eye ratio of female to male was 1.0 (48.5:45.5).^17^

FM is one of relatively common FSSs that overlap with others such as IBS, and autoimmune rheumatic diseases such as SS. Prevalence estimates for FM in patients with IBS range from 20% to 65% and the superiority of females in terms of FM frequency is known.^22^ In this study we found that FM rate was lower than the previously reported and all FM patients were women. Even though statistically insignificant, dry eye was higher in IBS patients without FM.

Antinuclear antibodies (ANA) are useful and important complementary tools for the diagnosis of patients with autoimmune diseases, but several physiological and pathological factors might favor the development of ANA in the non-rheumatic population, such as pregnancy, advanced age, family history of autoimmune disease and infectious, and also in patients with FM.^23^-^26^ While, the prevalence of ANA in the healthy population is estimated between 3-15%, there is a wide range in FM patients as 8.8-30% and in SS patients as 59-85%.^27^-^29^ In a study, Carroccio et al.^30^ reported ANA positivity and autoimmune diseases in 1 (2%) and 2 (4%) in the retrospective part and in 3 (6%) and 1 (2%) patient with IBS in prospective part of their study, respectively. However, authors didn’t provide the details about the autoimmune diseases in the IBS patients. In our study, ANA frequency was relatively higher than the reported by Carroccio et al.^30^ Of the 12 ANA positive-IBS patients, two patients were diagnosed as SS and three patients were diagnosed as FM. The remaining ANA positive patients did not show any signs of connective tissue disorders or FM. According to these results, the differential diagnosis between FM and connective tissue diseases should be done more carefully in these patients since ANA positive-IBS patients have SS and FM in comparable rates.

There are several limitations in this study. We used a cross-sectional approach and evaluated a relatively small patient group. In addition, we did not screen for other systemic diseases which have sicca syndrome and ANA positivity, such as autoimmune thyroid diseases.

In addition, no biopsy was performed in patients who had local or systemic disease that may cause dry eye but no clinical finding other than dry eye. SS can be present as a second cause for dry eye in such patients.

## CONCLUSION

Diagnosis of underlying systemic disease in IBS patients presenting with dry eye in particular is important. Specific diagnosis such as SS can facilitate more appropriate treatment and allow monitoring of potentially life-threatening complications. We recommend that all patients should be questioned for dryness of the mouth and eyes, and if necessary, evaluated for SS.
